# Composite Scaffolds Based on Intestinal Extracellular Matrices and Oxidized Polyvinyl Alcohol: A Preliminary Study for a New Regenerative Approach in Short Bowel Syndrome

**DOI:** 10.1155/2018/7824757

**Published:** 2018-05-27

**Authors:** Francesca Grandi, Elena Stocco, Silvia Barbon, Anna Rambaldo, Martina Contran, Francesco Fascetti Leon, Piergiorgio Gamba, Pier Paolo Parnigotto, Veronica Macchi, Raffaele De Caro, Andrea Porzionato

**Affiliations:** ^1^Department of Women's and Children's Health, Pediatric Surgery, University of Padua, Via Giustiniani 3, 35121 Padua, Italy; ^2^Section of Human Anatomy, Department of Neurosciences, University of Padua, Via Gabelli 65, 35121 Padua, Italy; ^3^Foundation for Biology and Regenerative Medicine, Tissue Engineering and Signaling (TES) ONLUS, Via De Sanctis 10, Caselle di Selvazzano Dentro, 35030 Padua, Italy; ^4^Department of Pharmaceutical and Pharmacological Sciences, University of Padua, Via Marzolo 5, 35131 Padua, Italy

## Abstract

Pediatric Short Bowel Syndrome is a rare malabsorption disease occurring because of massive surgical resections of the small intestine. To date, the issues related to current strategies including intestinal transplantation prompted the attention towards tissue engineering (TE). This work aimed to develop and compare two composite scaffolds for intestinal TE consisting of a novel hydrogel, that is, oxidized polyvinyl alcohol (OxPVA), cross-linked with decellularized intestinal wall as a whole (wW/OxPVA) or homogenized (hW/OxPVA). A characterization of the supports was performed by histology and Scanning Electron Microscopy and their interaction with adipose mesenchymal stem cells occurred by MTT assay. Finally, the scaffolds were implanted in the* omentum* of Sprague Dawley rats for 4 weeks prior to being processed by histology and immunohistochemistry (CD3; F4/80; Ki-67; desmin; *α*-SMA; MNF116).* In vitro* studies proved the effectiveness of the decellularization, highlighting the features of the matrices; moreover, both supports promoted cell adhesion/proliferation even if the wW/OxPVA ones were more effective (*p* < 0.01). Analysis of explants showed a continuous and relatively organized tissue wall around the supports with a connective appearance, such as myofibroblastic features, smooth muscle, and epithelial cells. Both scaffolds, albeit with some difference, were promising; nevertheless, further analysis will be necessary.

## 1. Introduction

Pediatric Short Bowel Syndrome (SBS) is a rare malabsorption condition with high morbidity and mortality rates in children [1–4]. It may be congenital [5–8], but more often it is the consequence of massive surgical resections of the small intestine [9, 10] ascribable to midgut* volvulus*, necrotizing enterocolitis, vascular thrombosis, mesenteric tumor, or abdominal trauma [11–13]. The loss of over 50%–75% of normal small bowel length results in a significantly decreased functional absorptive area of the intestine, which determines a broad diversity of metabolic and physiologic disturbances (i.e., weight loss, dehydration, and vitamin deficiencies) [11, 14–18]. To date, most patients are initially managed with total parental nutrition; nevertheless, over time, they may incur in high risk of catheter-related septicemia and parental nutrition-associated liver disease [18–20]. Other methods to treat SBS include surgical procedures such as longitudinal intestinal lengthening and tapering (LILT) or serial transverse enteroplasty (STEP) up to heterotopic small bowel transplantation but all of them have carried significant limitations and risks [21]. Thus, new treatment strategies should be searched and to this purpose the field of tissue engineering (TE) may be promising [22, 23].

As the severity of malabsorption depends on the residual bowel length, creating new bowel to increase intestinal length is a challenge that must be addressed. In 1997, Choi and Vacanti [24] first reported the development of a Tissue Engineered Small Intestine (TESI). It was manufactured employing multicellular aggregates derived from rat intestine and containing both mucosal and mesenchymal elements (i.e., organoid units (OUs)) which were seeded onto a porous synthetic biodegradable polymer scaffold made of polyglycolic acid (PGA). Since that moment, many other authors investigated the efficiency of the interaction between the intestinal OUs and the scaffolds. Supports, in the form of discs [25], or tubes were made of (a) PGA [25, 26], (b) copolymer [poly(lactic-co-glycolic acid)] (PLGA) [27], and (c) PGA coated with poly-L-lactic acid (PLLA) and Collagen Type I [11, 14, 16, 28–38]. Following seeding with intestinal OUs (thirty minutes up to an hour and a half later), the tubular scaffolds were implanted in the* omentum*. Interestingly, Lloyd and Colleagues [27] also described the lumen injection with the OUs after 4 weeks from the implantation. Thus, after a variable period (3 or 6 weeks), the neo-formed cyst-like structures were opened longitudinally and anastomized to jejunum [14, 28, 29, 31, 32, 39, 40] or to the large intestine [40] and the outcome was analyzed at different end-points up to 56 weeks [29] considering neomucosa growth and its architecture.

Despite the encouraging results with the use of OUs, difficult clinical scenarios could arise; in fact, a substantial quantity of healthy intestine is required to obtain an adequate number of OUs to be seeded onto the biodegradable polymer [17, 35]. Moreover, to date, the materials selection for TESI is restricted to PGA, PLGA, and PGA coated with poly-L-lactic acid (PLLA) and Collagen Type I. Interestingly, Boomer and Colleagues [41] investigated, in a preliminary study, the possibility of using other polymers (poly(ɛ-caprolactone) (PCL), poly(d-lactic acid-co-glycolic acid) (PDLGA), and polyurethane (PU)) but the formation of intestinal tissue was not considered, as the work aimed at a characterization of different scaffold materials to identify which one best suited TESI production from an histological, mechanical, and biodegradative point of view. The use of supports based on collagen rather than decellularized intestinal extracellular matrix (ECM) was analyzed [42–44] but, to our knowledge, the combination with a synthetic biomaterial was never reported in literature.

To overcome the limitations in the panorama of TESI, the aim of this study was to preliminarily investigate the potentiality of a novel composite scaffold for TESI without resorting to OUs. In particular, we manufactured a support that combines the biological features of intestinal ECM (considered as whole or homogenized/lyophilized) with the mechanical properties of a novel biocompatible and resorbable hydrogel developed by our research group, that is, 1% oxidized polyvinyl alcohol (OxPVA). OxPVA derived from oxidation of polyvinyl alcohol (PVA) by potassium permanganate in diluted perchloric acid [45]. Oxidation allows reengineering the polymer, improving its degradation and drug-release properties after cross-linking by freeze-thawing. To date, we experienced the suitability of OxPVA-derived hydrogels for the manufacture of cartilage scaffolds as well as for their drug-delivery properties. OxPVA scaffolds were loaded with a model protein (i.e., bovine serum albumin (BSA)) or bioactive growth factors (i.e., tumor necrosis factor-*β* [45]; transactivator transduction domain-ciliary neurotrophic factor [46]). Intrinsic properties of the scaffolds vary along with the oxidation degree; hence, modulating the stoichiometry of the oxidative reaction, it is possible to obtain PVA-derived polymers that can be used to prepare scaffolds with different biodegradative and bioactive properties in accordance with the tissue to be regenerated. This peculiar characteristic gives to OxPVA the ability to mimic different types of tissues, being extremely interesting for tissue engineering purposes.

## 2. Materials and Methods

### 2.1. Scaffolds Preparation

Two different composite scaffolds were prepared. Those were based on 1% OxPVA combined with decellularized intestinal mucosa as a whole (wW/OxPVA) or homogenized (hW/OxPVA). Scaffolds of OxPVA were used as control.

#### 2.1.1. Sampling and Decellularization of Intestinal Extracellular Matrix

All animal procedures were approved by the ethical committee of Padua University, in agreement with the guidelines of the Italian Department of Health.

Adult Sprague Dawley rats, weighing approximately 250–350 g, were euthanized by carbon dioxide asphyxiation. After shaving the abdomen, a middle incision extending from the xyphoid process to the symphysis* pubis* was performed. Afterwards, the small intestine was eviscerated, dissolving the adhesions to the large intestine, and the stretch ranging from the ligament of Treitz to the* caecum* was taken. After washing carefully with PBS supplemented with a 2% solution of penicillin and streptomycin, decellularization occurred according to the detergent-enzymatic method by [47]. Briefly, samples were soaked in distilled water for 72 h at 4°C, changing the aqueous solution every 2 h, 4% sodium deoxycholate for 4 h at room temperature (RT), and 2,000 KU (Kunitz Units) DNase-I in 1 M NaCl for 2 h at RT. The procedure was repeated for 2 times and the effectiveness of the procedure was evaluated through histological analysis.

#### 2.1.2. Histological Analysis

Specimens of decellularized small intestine were soaked in cold isopentane, frozen in liquid nitrogen fumes, and maintained at −80°C for 24 h. Thereafter ice-included fragments were sliced in 4 *μ*m slices using a cryomicrotome (Leica CM 1850 UV). The slices were fixed with acetone and mounted with VECTASHIELD mounting medium for fluorescence with DAPI (Vector Laboratories, Burlingame, CA, USA). In parallel, decellularized samples were fixed in 10% formalin solution in neutral PBS, paraffin-embedded, and stained also with hematoxylin and eosin according to routine protocols.

#### 2.1.3. Preparation of Intestinal Matrices

The decellularized small intestine was carefully dried using a sterile gauze to remove excess water. To obtain wW, the tissue was carefully laid down into a culture plate and then opened longitudinally, turning the* lumen* outwards. Thereafter, 1 cm long samples were cut using a surgical blade and frozen at −20°C to make them more manageable. In parallel, hW was also prepared as previously described [48]. Briefly, a preweighted quantity of decellularized ECM (1 gr) was soaked with 15 mL of 10% acetic acid solution (2.5 M) in deionized water (dH_2_O) and homogenized at 0°C using Ultra-Turrax homogenizer (Janke & Kunkel GmbH, Staufen, Germany) 8 times/20 sec with intervals of 5 min. Then, a volume of 3,5 mL of the obtained suspension was poured into moulds (Petri dishes, diameter: 60 mm) and frozen at −20°C overnight before lyophilizing.

#### 2.1.4. Polymer Preparation and Manufacture of the Composite Scaffold

Scaffolds were prepared combining wW and hW, respectively, with a polymeric solution of OxPVA, which was obtained according to a controlled chemical oxidation [45]. Thus, composite scaffolds were prepared, pouring a volume of 3,5 mL of OxPVA into a P60 culture plate and setting down carefully the wW and hW, respectively, prepared as previously described; the wW was laid with the villi facing out. Thereafter, the hydrogel and the ECMs were physically cross-linked according to a freezing-thawing (FT) process, which allowed embedding the matrix upon the scaffold and obtaining the cryogel. The FT process consisted of 7 cycles of freezing at −20°C and thawing at −2.5°C. At the end of the treatment, composite scaffolds were kept at −20°C until use. Discoidal samples with a diameter of 0.7 mm were then obtained from each membrane using a punch. Scaffolds made of OxPVA were used as control.

#### 2.1.5. Morphological Characterization of Scaffolds

After manufacture, OxPVA and composite scaffolds were investigated for their morphology through H&E staining. In parallel, the superficial ultrastructure of the supports was analyzed by a Scanning Electron Microscope (SEM). Samples were fixed with 2.5% glutaraldehyde in 0.1 M cacodylate buffer (pH 7.2) for 24 h and dehydrated using a graded ethanol series. Afterwards, critical point drying and gold sputtering occurred prior to observing supports using a SEM (Stereoscan-205 S; Cambridge Instruments, Pine Brook, NJ, USA).

### 2.2. In Vitro Assessment of the Biological Properties of Scaffolds

#### 2.2.1. Cell Cultures

Primary human adipose mesenchymal stem cells (Ad-MSCs) were purchased from tebu-bio SAS (France, Europe) and they were thawed and expanded according to the manufacturer's instructions. The proliferative medium consisted of *α*MEM (alpha-modified Eagle's medium without nucleosides), 15% fetal bovine serum (FBS), and 1% antibiotic solution (Thermo Fisher Scientific, Waltham, MA, USA). The culture medium was refreshed every 2 days. Cell cultures were observed daily by optical microscope DM/IL (Leica), and pictures were taken using a Nikon Digital Sight Ds-SMCc camera (Nikon Corporation).

#### 2.2.2. Ad-MSCs Culture on Scaffolds

Scaffolds were disinfected by 4 washes of 2 h each in PBS supplemented with 2% antibiotic solution; thereafter, they were incubated overnight at 37°C in basal medium. Subsequently, Ad-MSCs at 80% of confluence were detached from the culture plate by treatment with Trypsin/EDTA, centrifuged at 1500 rpm for 5 min, and resuspended in complete proliferation medium. Each support was seeded with 20,000 cells/cm^2^ in complete proliferative medium. In parallel, cells were also seeded in a 48-well plate as positive control.

#### 2.2.3. Evaluation of Ad-MSCs Behavior on Scaffolds

At 7 days from seeding, the proliferative activity of cells on OxPVA, wW/OxPVA, and hW/OxPVA scaffolds was evaluated using (4,5-dimethylthiazol-2-yl)-2,5-dimethyl tetrazolium bromide (MTT) (0.5 mg/mL) for 4 h. The resulting precipitates of formazan were dissolved by 2-propanol acid (0.04 M HCl in 2-propanol). Microplate autoreader EL 13 was used to measure the optical density of the solution at 570 nm. Results were expressed as number of cells grown on seeded surface.

### 2.3. In Vivo Behavior of Scaffolds

#### 2.3.1. Surgery: Implant and Explant of Scaffolds

Fifteen Sprague Dawley rats were randomly divided into 3 experimental groups: OxPVA, *n* = 5; wW/OxPVA, *n* = 5; and hW/OxPVA, *n* = 5. After anaesthesia with isoflurane/oxygen, their abdomen was carefully shaved and a middle incision extending from the xyphoid process to the symphysis* pubis* was performed. The scaffolds (one for each animal) were allocated with transfected sutures (monofilament, PDS 4/0) in the* omentum *and the abdominal wall was then synthesized by layers through stitches in resorbable filament (Novosyn 4/0 and Monosyn 4/0). Soon after surgery, the rats were given anti-inflammatory Rimadyl (0.5 mg/kg) and antibiotic Baytril (5 mg/kg) therapy for 5 days and were allowed to recover in the cage. Four weeks after surgery, rats were euthanized by overdose of gaseous CO_2_. The supports were identified, carefully removed, and treated for further macroscopic evaluations and histological/immunohistochemical analysis.

#### 2.3.2. Histological and Immunohistochemical Analysis

Explanted scaffolds were fixed with a solution of 10% formalin in neutral PBS and paraffin-embedded. Thereafter, serial sections of 5 *μ*m in thickness were cut, dewaxed, and rehydrated according to routine protocols before staining with haematoxylin and eosin (H&E). In parallel, immunological characterization was also performed with Dako Autostainer/Autostainer Plus (Dako). The following antibodies diluted in EnVision^TM^ FLEX were used: anti-CD3 (polyclonal rabbit anti-human CD3, A 0452; Dako, Milan, Italy) diluted 1 : 1000; rabbit anti-mouse anti-F4/80 (sc-26643-R; Santa Cruz Biotechnology, CA, USA) diluted 1 : 800; monoclonal rabbit anti-human Ki-67 (M3060; Spring Bioscience, UCS Diagnostic, Rome, Italy) diluted 1 : 200; polyclonal rabbit anti-desmin (29593; AnaSpec, San Jose, California, USA) diluted 1 : 400; mouse monoclonal anti-*α*-SMA (A2547; Sigma, Missouri, USA) diluted 1 : 500; and monoclonal mouse anti-human cytokeratin clone MNF116 (M0821; Dako) diluted 1 : 200. After antigen unmasking, the sections were incubated with peroxidase-blocking serum (EnVision FLEX Peroxidase-Blocking Reagent; Dako) for 5 min in order to remove the nonspecific binding and then for 30 min with the primary antibody. Sections treated with anti-*α*-SMA, anti-desmin, anti-Ki-67, and anti-MNF116 antibodies were then incubated with the secondary antibodies for 15 min (EnVision FLEX Mouse-Linker and EnVision FLEX Rabbit-Linker; Dako). For all protocols, EnVision FLEX/HRP polymer was used for 20 min and 3,3′-diaminobenzidine (EnVision FLEX Substrate Buffer + DAB + Chromogen; Dako) was used in order to highlight the positivity of the reaction. All sections were finally counter-colored with hematoxylin (EnVision FLEX, Hematoxylin, Dako) for 5 min in order to mark cell nuclei, dehydrated with a descending alcohol scale, xylene, and mounted. All the steps were performed at RT.

### 2.4. Statistical Analysis

Statistical analyses were performed by the Kruskal-Wallis test and Dunn's multiple comparison test. Results were expressed as mean ± standard deviation (SD). *p* < 0.05 was considered to be statistically significant. Statistical calculations were carried out by Prism 3.0.3 (GraphPad Software, San Diego, CA).

## 3. Results and Discussion

### 3.1. Preparation and Characterization of the Matrices

In TE, the use of decellularized matrices gained special attention owing to their clinical success in tissue reconstruction and their advantage of preserving the native architecture of the tissue along with the ECM [49, 50]. Indeed, the identification of the adequate decellularization protocol is a challenge, as many methods do not allow maintaining both the mechanical properties and inherent biofactors [42, 51]. Recently, we used biological matrices derived from homogenization and lyophilization of decellularized tissues, and we stressed the potentiality of these innovative biomaterials, which take advantage of ECM macromolecules more than their macro- and microarchitecture [48, 52]. Thus, in the present work, we decided to evaluate ECM eventual efficacy also in intestine TE by comparing two scaffold types derived from decellularized intestinal wall (whole and homogenized).

**Figure 1:**
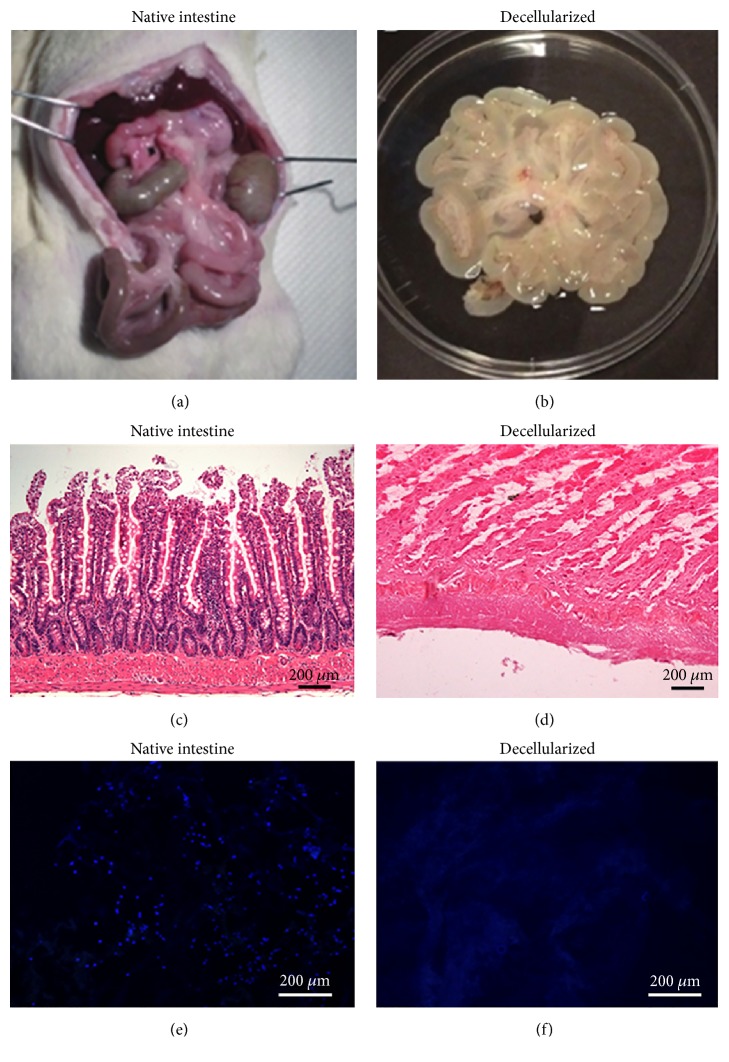
Native and decellularized rat small intestine. Gross appearance ((a) and (b)), hematoxylin/eosin ((c) and (d)), and DAPI staining ((e) and (f)) of small intestine before ((a), (c), and (e)) and after ((b), (d), and (f)) decellularization. Scale bar: 200 *μ*m.

Decellularization process produced progressive whitening of the intestine, which at the end of the process appeared translucent and partially transparent, although maintaining its anatomic recognizability (Figure 1), also in accordance with previous literature on the matter [43]. A certain loss of mechanical properties was obviously found, with tendency of the intestinal lumen to collapse; notwithstanding, cautious manipulation of the bowel was still possible.

Decellularized segments of small intestine were histologically analyzed in comparison with native tissue (Figure 1). Our decellularization protocol guaranteed good removal of cell materials, with quite good preservation of the three-dimensional architecture of the extracellular matrix. DAPI staining demonstrated the total absence of nuclei in the decellularized tissue, confirming the efficacy of the treatment in completely removing the immunogenic cellular components. Profiles of villi and intestinal glands were still clearly recognizable, although in the absence of epithelial layer. Residues of the serosal layer and mesenteric insertion were partially identifiable on the outer side. Thus, our results were not in complete agreement with Patil and colleagues [42] who preferred the actions of DMSO together with Triton X-100, claiming that intraluminal decellularization of the small intestine by sodium deoxycholate/DNase does not give satisfactory results.

### 3.2. Composite Scaffolds

Following the production of decellularized intestinal ECMs (wW and hW) and OxPVA, composite bioscaffolds were prepared by physical cross-linking through FT process. Very few studies about the fabrication of synthetic polymer/ECM composites with high resilience and large strain have been reported in the literature [53]. Nevertheless, in the conditions considered, the presence of a sustain platform is a prerequisite as both the wW and the hW were not consistent enough to maintain their shape. Moreover, the bioscaffold must be firm enough to be enveloped by the* omentum* without folding and altering its structure [11]. Hybrid composites containing synthetic polymers with high mechanical strength and naturally derived components, which create a biomimetic environment, are actually one of the most promising biomaterials [53].

**Figure 2:**
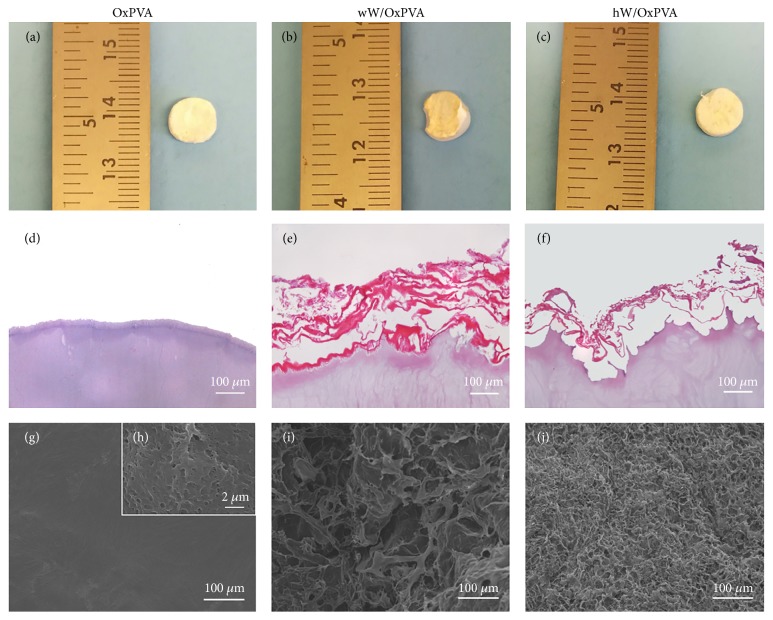
Macroscopic, microscopic, and ultrastructural appearance of scaffolds. Gross appearance ((a)–(c)), hematoxylin/eosin staining ((d)–(f)), and SEM micrographs ((g)–(j)) of OxPVA and composite scaffolds (wW/OxPVA; hW/OxPVA). Scale bar: 100 *μ*m; scale bar in upper right insert (h): 2 *μ*m.

As previously discussed, the ECM was considered in two variants: whole and homogenized. This allowed comparing the biological role of the protein matrix (hW) and the involvement of the structural architecture (wW). The composite scaffolds obtained by cross-linking are shown in Figure 2. According to histological analysis, the three-dimensional architecture of intestinal wall was partially preserved in the composite wW/OxPVA scaffolds; as regards the decellularized/homogenized intestinal wall, it appeared like a thin protein coating in the hW/OxPVA scaffolds. The ECM showed different thickness in the two composite supports, with wW being thicker than hW. In all samples, the OxPVA layer was clearly recognizable, with regular profile. In parallel, ultrastructural analysis by SEM confirmed the data from histology. Both wW/OxPVA and hW/OxPVA surfaces showed a spongy appearance with thinner texture in wW/OxPVA. OxPVA appeared like a surface characterized by a certain rugosity, which was appreciable only at high magnification.

#### 3.2.1. Interaction between Composite Scaffolds and Ad-MSC Cultures

The ultimate goal of the present study involved the implantation of the composite scaffolds as such without a preseeded cellular component. Nevertheless, to preliminarily evaluate their biological properties, cell culture assays with AD-MSCs were performed.

**Figure 3:**
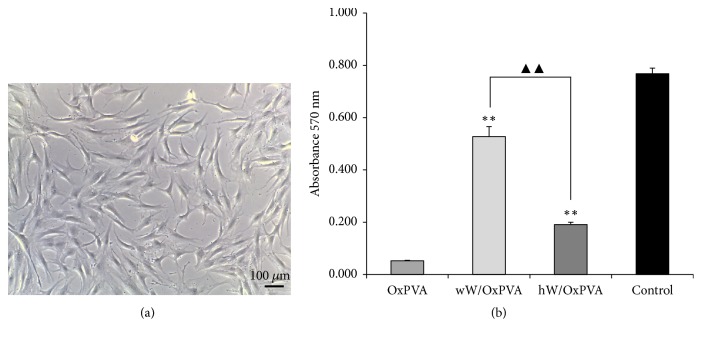
Ad-MSCs-scaffolds interaction. (a) Human Ad-MSCs at optical microscope (scale bar: 100 *μ*m). (b) Evaluation by MTT assay of cell proliferation on OxPVA, wW/OxPVA, and hW/OxPVA at 7 days from seeding (^*∗∗*^*p* < 0.01, wW/OxPVA and hW/OxPVA versus OxPVA; ^▲▲^*p* < 0.01, wW/OxPVA versus hW/OxPVA).

Despite TESI progress, limitations remain in the use of autologous intestinal cells. Namely, (a) a large number of OUs are required to generate even a modest amount of neomucosa [25] and in patients with diseased or damaged bowel isolating OUs may be extremely difficult or even impossible; (b) the donor tissue is difficult to expand [54]. These drawbacks prompted the research towards an alternative cell source. At present, intestinal stem cells are considered the only valid option because of their ability to differentiate in all types of intestinal cells. Nevertheless, the isolation of pure stem cells from intestinal crypts is also difficult because of the lack of specific stem cells markers [55]. Hence, in the present work, we focused on mesenchymal stem cells, in particular Ad-MSCs, which are currently considered an interesting cell source for tissue engineering of epithelium [56], endothelium [57–59], and smooth muscle, particularly for pediatric applications [60, 61]. In Figure 3(a), the fibroblastoid appearance of Ad-MSCs is shown. At 7 days from seeding, proliferating cells on both composite scaffolds were significantly more numerous compared to OxPVA supports (*p* < 0.01) (Figure 3(b)). This is consistent with previous works by our group showing low cellular adhesion properties for this material [45], probably due to excessive hydrophilicity of the support, as well as inadequate porosity. In addition, the proliferation assay showed that although the macromolecular component of hW was able to support cell adhesion and proliferation, the wW showed significantly better results in terms of adhesion and proliferation (*p* < 0.01). It was possible to state that matrix architecture is a further stimulus, in addition to that of the specific protein components, to recreate a microenvironment suitable for the vitality and proliferation of Ad-MSCs.

#### 3.2.2. Evaluation of In Vivo Behavior of Scaffolds

Many studies suggest that artificial matrices alone may provide instructive cues for retrieving stem cells from the surrounding tissues; hence, we decided to also implant native OxPVA for comparison. In our experimental protocol, OxPVA, wW/OxPVA, and hW/OxPVA scaffolds were not placed in direct continuity with the bowel but sutured in the omental context as the peritoneal microenvironment is known to play an important role in the tissue engineering of small intestine [41].

**Figure 4:**
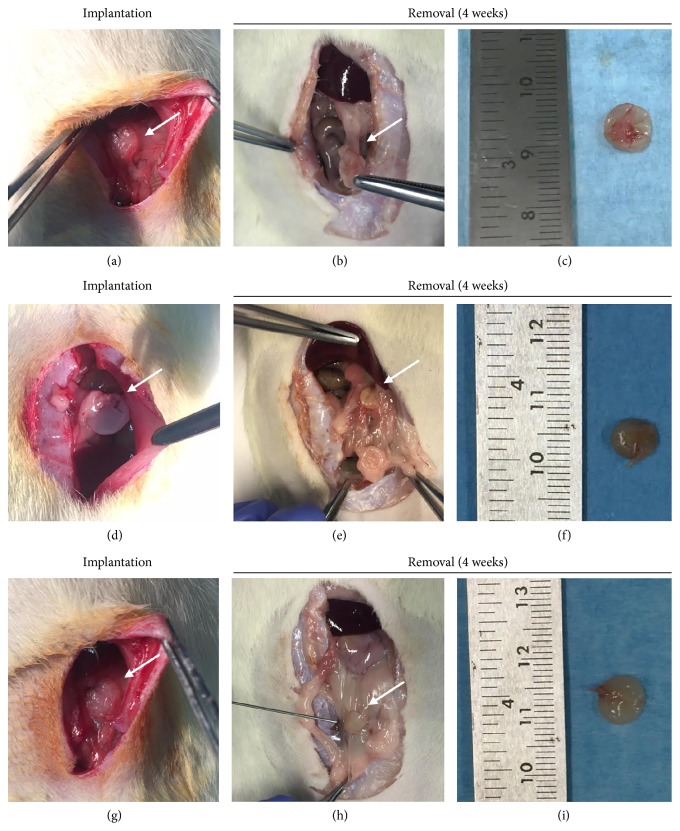
*In vivo* behavior of scaffolds. Implantation and removal of OxPVA (control) ((a)–(c)), wW/OxPVA ((d)–(f)), and hW/OxPVA ((g)–(i)) from the omentum of Sprague Dawley rats.

**Figure 5:**
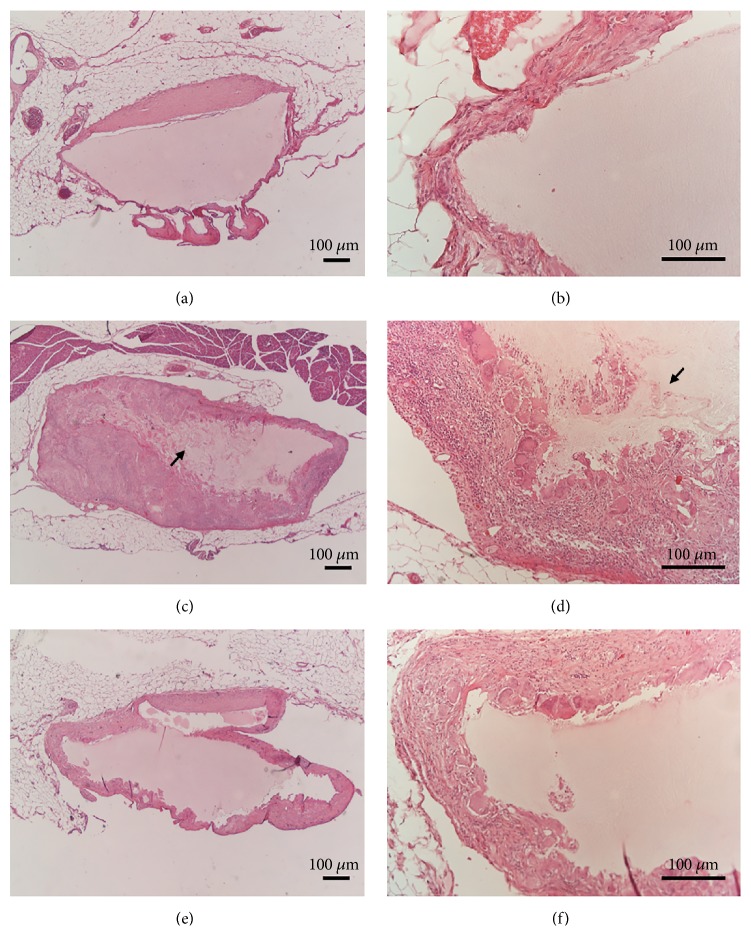
Histological characterization of explants. Hematoxylin/eosin staining of OxPVA ((a) and (b)), wW/OxPVA ((c) and (d)), and hW/OxPVA ((g)–(i)) supports. Degradation features are more evident in the wW/OxPVA support than in hW/OxPVA and OxPVA scaffolds. The peripheral areas of wW/OxPVA scaffold in which the polymer appears less dense and continuous are indicated in (c) and (d) by the black arrows. Note also the reduction in scaffold size (wW/OxPVA) (scale bars: 100 *μ*m).

After surgery, no animal showed surgical site infections or was euthanized because of complications. At the time of explantation (week 4), all scaffolds were still recognizable and no inflammatory reaction was observed (Figure 4). Four weeks is a shared end-point by many authors to observe the first development stages of the TESI [11, 16, 25–27, 34–36]. After this period, OxPVA was still present and recognizable in each type of scaffold. Partial reabsorption aspects were identifiable in the most superficial portions as confirmed by microscopic examination; the degradation aspects were more evident in the wW/OxPVA scaffolds than in hW/OxPVA and OxPVA ones (Figure 5).

Ideally, the degradation rate of scaffolds should be slow enough to sustain the proliferation and differentiation of cells eventually seeded or migrated towards the support while promoting the production of new ECM without restricting the eventual formation of new tissue [41]. Interestingly, the obtained results seemed to confirm a proper degradation rate by OxPVA (Figure 5).

According to the literature, most authors worked with tubular-like scaffolds resembling the natural gross appearance of the small intestine to develop a TESI. Otherwise, similarly to our research study, Wulkersdorfer et al. [25] manufactured disc-shaped supports (PGA/Matrigel seeded with OUs), observing a neomucosal growth with a certain complex architecture and endothelial cells after 4 weeks into the subcutaneous tissue of Lewis rats; these data suggest the potential adequacy of the discoidal scaffold to address the aim of the work. In all the samples, inflammatory reactions were not present, assessing the biocompatibility of the biomaterial as well as the suitability of the decellularization protocol for the composite scaffolds. Each scaffold type was surrounded by a continuous and relatively well-organized tissue that showed higher thickness in the wW/OxPVA; the outer layer had a connective-like appearance.

**Figure 6:**
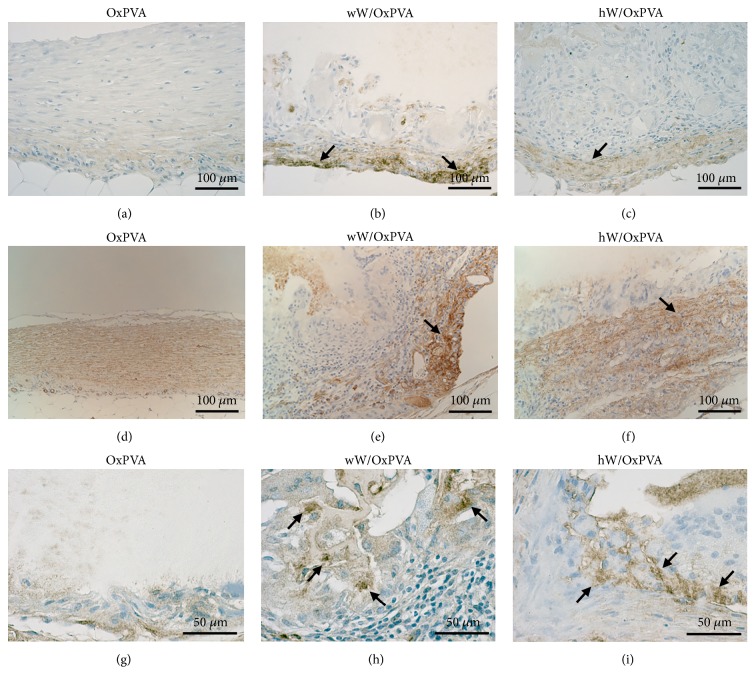
Immunohistochemical characterization of explants. Localization of cells positive to desmin ((a)–(c)), *α*-SMA ((d)–(f)), and MNF116 ((g)–(i)) in OxPVA, wW/OxPVA, and hW/OxPVA samples at 12 weeks from surgery. Moderate anti-desmin and anti-*α*-SMA immunoreaction (black arrows) is recognizable in the outer layers of the tissue surrounding the wW/OxPVA and hW/OxPVA scaffolds, indicating myofibroblastic and/or smooth muscle cell differentiation. Conversely, such positive staining is absent (desmin) or scant (*α*-SMA) in tissues around OxPVA. Partial positivity for the pan-cytokeratin marker MNF-116 is shown in cells in the inner layer of the tissue surrounding the wW/OxPVA and hW/OxPVA scaffolds (black arrows) (scale bar: ((a)–(f)) 100 *μ*m; ((g)–(i)) 20 *μ*m).

In hW/OxPVA and wW/OxPVA, immunohistochemical analyses gave partial positivity for desmin and *α*-SMA, indicating myofibroblastic and/or smooth muscle cell differentiation; conversely, these immunoreactions were very faint or negative in the tissue surrounding native OxPVA (Figure 6).

**Figure 7:**
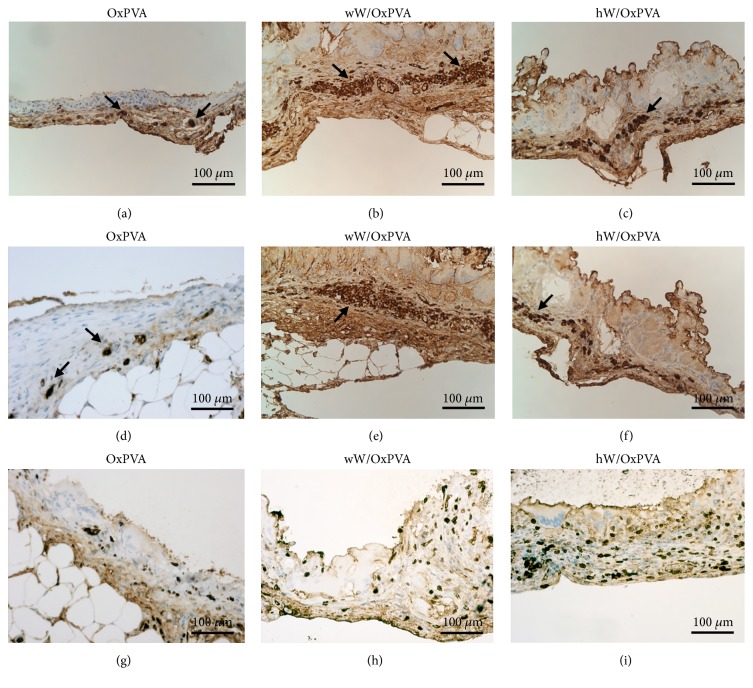
Immunohistochemical characterization of explants. Localization of cells positive to CD3 ((a)–(c)), F4/80 ((d)–(f)), and Ki-67 ((g)–(i)). Immunoreactive lymphocytes (CD3) and macrophages (F4/80) are detectable in the external layer of tissue around wW/OxPVA and hW/OxPVA, as shown by the black arrows. Conversely, there are no inflammatory elements in the inner layer. Cell proliferation rate (Ki-67 positive cells) is higher in the tissue surrounding wW/OxPVA and hW/OxPVA scaffolds than OxPVA ones (scale bar: 100 *μ*m).

Cubic or cylindrical cells, with basal nuclei, disposed in layers or glandular-like patterns were appreciable on the inner side adjacent to the OxPVA, in both the wW/OxPVA and hW/OxPVA. The epithelial nature of these cells was confirmed by immunohistochemical analyses, which were negative for desmin, *α*-SMA (Figure 6), CD3, and F4/80 (Figure 7), and partially positive for pan-cytokeratin marker MNF116 (Figure 6). In some fields, the invaginations of the epithelial layer, of various depth and extent, acquired a clear crypt-like appearance, although differentiation in specialized (muciparous, enteroendocrine, and Paneth) cells was not found. Villous-like structures were not clearly recognizable. Epithelial components were not observed in the tissue surrounding the native OxPVA.

Considering the experimental works of other authors in the development of TESI models, at 4 weeks from implantation, Sala et al. [16] described a TESI with a desmin and an *α*-SMA-positive muscularis; moreover, intestinal subepithelial myofibroblasts negative for desmin and positively stained for SMA were identified just below the base of the crypt epithelium. Similar findings were highlighted also by Levin et al. [11]. With respect to our study, Sala et al. [16] and Levin et al. [11] worked with tubular scaffolds made of PGA coated with PLLA and Collagen Type I, which were seeded with OUs before the implant in* omentum*; conversely, the end-point and the implantation site were the same.

Lymphocytes (CD3^+^ cells) and macrophages (F4/80^+^ cells) were mainly found in the outer layer of the tissue surrounding wW/OxPVA and hW/OxPVA, whereas the inner epithelial layer did not show any inflammatory element. The connective tissue surrounding OxPVA also showed rare CD3^+^ or F4/80^+^ cells (Figure 7).

Vascular structures with organized endothelium were also detectable in the outer layer of the tissue surrounding wW/OxPVA and hW/OxPVA and in the connective tissue surrounding OxPVA, consistently with local neoangiogenesis.

The inductive stimuli of scaffolds on cell proliferation were also evaluated by immunohistochemical localization of the Ki-67 nuclear antigen. Apart from local proliferation of lymphomonocytic cells, moderate cell proliferation was found in the epithelial and myofibroblastic/smooth muscle cells surrounding wW/OxPVA and hW/OxPVA; lower proliferation rate was appreciable in the connective tissue surrounding native OxPVA (Figure 7). Moreover, Torashima et al. [36] evaluated the presence of Ki-67 positive elements to quantify epithelial proliferation after 4 weeks of implantation in the* omentum* of a PGA-based scaffold seeded with OUs.

One of the most important findings of this preliminary study concerns the potentials of the hybrid scaffolds manufactured combining a novel synthetic biodegradable biomaterial, that is, oxidized polyvinyl alcohol, with the small intestine-derived ECMs. Beyond confirming the versatility of the polymer for tissue engineering applications, the* in vitro *and* in vivo* data suggested the interesting role exerted by the lyophilized ECM. Even if the importance of the tissue-architecture's maintenance is a matter of fact, the discovery of an alternative approach in using ECMs is particularly interesting. By virtue of the complexity of proteins and growth factors by which matrices are made of, their biologic activity is more adequate than that of scaffolds prepared using natural polymers alone. Hence, to be able to exploit this peculiarity is an important achievement.

## 4. Conclusions

As assessed by the histological and immunohistochemical analysis performed on explanted samples, both wW/OxPVA and hW/OxPVA scaffolds did not induce the differentiation of muciparous, enteroendocrine, and Paneth cells; moreover, villous-like structures were also not yet recognizable. Further research will consider the manufacture of tubular biohybrid scaffolds, prolonged end-points, and a direct anastomosis with the small intestine in order to increase the differentiation of the structural elements of the intestinal mucosa. Thus, the aim will be to optimize the manufactured scaffolds, obtaining a tissue substitute resembling the complex histological features of the small intestine. Hence, varying the discoidal scaffold shape into a tubular one, as well as adjusting the experimental conditions, may be helpful in the development of an adequate tissue-engineered platform supporting a more complex tissue regeneration, in accordance with the characteristic histological features of the small intestine.
